# Assessment of postoperative prognosis in patients with acute ST-segment elevation myocardial infarction after PCI using LRP1

**DOI:** 10.3389/fcvm.2025.1520696

**Published:** 2025-02-27

**Authors:** Jingwen Guan, Yikang Xu, Limin Liu, Mengran Su, Jingru Ma

**Affiliations:** Cardiology Department, The Second Affiliated Hospital of Shenyang Medical College, Shenyang, China

**Keywords:** low-density lipoprotein receptor-related protein-1 (LRP1), acute ST-segment elevation myocardial infarction (STEMI), major cardiovascular ad verse events (MACE), prognosis, MACE

## Abstract

**Purpose:**

To evaluate the prognostic value of Low-density lipoprotein receptor-related protein 1 (LRP1) in patients with acute ST-segment elevation myocardial infarction (STEMI) following percutaneous coronary intervention (PCI).

**Method:**

This prospective study included 96 STEMI patients who underwent PCI and 19 control subjects with normal coronary arteries. Coronary blood was taken from both groups, and LRP1 expression levels were quantified using real-time quantitative PCR (qPCR). The STEMI patients were stratified into low, middle, and high LRP1 groups based on tertiles of LRP1 expression. The primary endpoint was the occurrence of major adverse cardiovascular events (MACE) during a six-month follow-up period post-PCI.

**Results:**

LRP1 expression in arterial blood was significantly lower in the STEMI group [0.63(0.23,1.1)] compared to the control group [1.5(0.84,1.85)] (*P* < 0.05). The incidence of MACE showed an increasing trend across the LRP1 tertiles: 6.7% (95% CI: 1.9–21.3%) in the low LRP1 group, 22.6% (95% CI: 11.4–39.8%) in the middle LRP1 group, and 41.9% (95% CI: 26.4–59.2%) in the high LRP1 group. The high LRP1 group exhibited a significantly higher MACE rate compared to the low LRP1 group (*P* < 0.05). Spearman's rank correlation analysis revealed positive correlations between LRP1 and both NT-proBNP and cTnT (*r* = 0.349, 95% CI: 0.156–0.515, *P* < 0.001; *r* = 0.328, 95% CI: 0.133–0.497, *P* = 0.001, respectively), and a negative correlation with LVEF values (*r* = −0.285, 95% CI: −0.460 to −0.087, *P* = 0.006). Receiver operating characteristic (ROC) analysis identified an LRP1 expression threshold of 0.79 for predicting MACE within six months post-PCI, with a sensitivity of 81.8% (95% CI: 61.5–92.7%), a specificity of 70% (95% CI: 58.5–79.5%), and an area under the curve (AUC) of 0.789 (95% CI: 0.688–0.890, *P* < 0.001).

**Conclusion:**

LRP1 expression appears to be an independent predictor of MACE in STEMI patients and may have prognostic value for short-term outcomes following PCI.

## Background

Acute ST-segment elevation myocardial infarction (STEMI) remains a critical cardiovascular emergency associated with significant morbidity and mortality. While the widespread implementation of percutaneous coronary intervention (PCI) has significantly reduced mortality rates over the past four decades (1, 2), a subset of patients still experience complications such as arrhythmias, heart failure, cardiogenic shock, and cardiac rupture post-PCI, adversely affecting their prognosis. Early identification of high-risk patients could facilitate more tailored treatment strategies and potentially improve outcomes.

Low-density lipoprotein receptor-related protein 1 (LRP1) has emerged as a molecule of interest in cardiovascular research. Studies have demonstrated that LRP1 plays a crucial role in the formation and progression of coronary atherosclerosis. In animal models, LRP1 agonists have shown promise in cardioprotection. For instance, the synthesized LRP1 agonist (SP16) has been found to rapidly induce Akt phosphorylation, exert anti-inflammatory effects, and inhibit programmed death of myocardial cells, thereby protecting the surviving myocardium adjacent to the infarction scar (3–5).

However, the potential of LRP1 as a prognostic marker in STEMI patients undergoing PCI remains largely unexplored. The complex role of LRP1 in the pathophysiology of myocardial infarction, involving processes such as inflammation, cell survival, and tissue remodeling, suggests that its expression levels may provide valuable insights into patient outcomes.

This study aims to investigate the relationship between LRP1 expression and myocardial ischemia, and to evaluate its potential as a prognostic marker in STEMI patients undergoing PCI. The findings may not only contribute to a better understanding of LRP1's role in STEMI but also provide a theoretical basis for the potential application of LRP1 agonists in the clinical management of STEMI patients.

## Materials and methods

### Study population and subgroups

This prospective observational study was conducted at the Second Hospital Affiliated to Shenyang Medical College from November 2022 to July 2023. The experimental group comprised 96 patients with their first diagnosis of STEMI who underwent emergency PCI within 12 h of symptom onset. Additionally, 19 patients with normal coronary angiograms hospitalized during the same period were selected as the control group. In the experimental group, there were 84 males and 12 females, with an age range of 31–86 years old and a mean age of 59. All methods were performed in accordance with the relevant guidelines and regulations.

Inclusion criteria: (1). Age ≥18 years. (2). STEMI based on: According to the fourth edition of the “Global Definition of Myocardial Infarction” (6, 7), the diagnosis of STEMI needs to meet the criteria of acute myocardial injury (elevated serum cTnT) and new ischemic electrocardiographic changes (ST-segment elevation) at the same time. (3). complete clinical data and biochemical examination data. (4). Complete clinical data and biochemical data. (5). Volunteer to participate in the clinical trial and sign the informed consent for enrollment. Exclusion criteria: (1). Previous old myocardial infarction, chronic heart failure, heart valve disease, myocarditis, dilated cardiomyopathy, hypertrophic cardiomyopathy, and congenital heart disease. (2). Suffering from Alzheimer's disease, malignant neoplasm, rheumatoid immune disease, thyroid disease, acute cerebrovascular disease, and psychiatric disease. (3). Combined with acute infections. (4). Recent application of glucocorticosteroids and antibiotics. (5). Combined with severe hepatic and renal function. (6). Combined with severe hepatic and renal insufficiency.6.Critically ill patients with multiple organ failure. (7). Patients with missing clinical information and uncooperative.

## Research methodology

### Clinical data collection

The general information of the admitted patients was collected, including age, sex, height, weight, medical history of hypertension, diabetes mellitus, family history of coronary heart disease, smoking history, systolic pressure, diastolic pressure, heart rate, blood routine, renal function, fasting blood glucose, blood lipids, N-Terminal pro-brain natriuretic peptide (NT-proBNP), Cardiac troponin T (cTnT), number of branches of coronary vascular disease, and the use of medications during hospitalization, as well as body mass index (BMI), mean arterial pressure and Gensini score, left ventricular ejection fraction (LVEF) from cardiac ultrasound.

### LRP1 expression measurement

**Figure 1:**
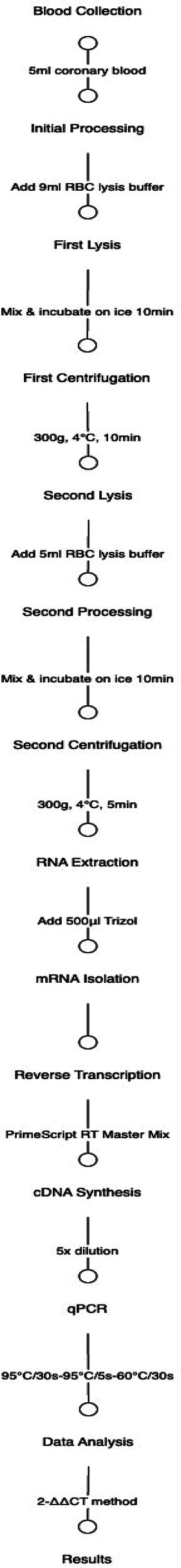
Workflow for LRP1 expression measurement in coronary blood samples.

LRP1 expression was measured in leukocytes isolated from coronary blood, with monocytes being the predominant source of LRP1 expression among white blood cells. Blood sampling was performed during coronary angiography prior to any intervention. For STEMI patients, approximately 5 ml of coronary blood was collected from the culprit vessel through a 6F guiding catheter after passing the lesion but before balloon dilation or stent implantation. For control subjects, blood was collected from the left anterior descending coronary artery using the same catheter system. All samples were immediately transferred to EDTA anticoagulation tubes and stored at −80°C until analysis. The sampling location was carefully documented in the catheterization report for each patient. The blood samples were immediately stored at −80°C for subsequent testing. The testing process was as follows: The complete workflow for LRP1 expression measurement is illustrated in Figure 1. 1 ml of blood sample and 9 ml of erythrocyte lysate were added to a 15 ml centrifuge tube → mix up and down ten times→ stand on ice for 10 min, during which time up and down for five times every 3min→ centrifugation at 300 g for 10 min at 4℃ → add 5 ml of erythrocyte lysate→ mix up and down for ten times→ stand on ice for 10 min, during which time up and down for five times every 3 min→ stand on ice for 10 min, during which time up and down for five times every 3 min → 4℃, centrifugation at 300 g for 5 min→centrifugation at 300 g for 5 min. Centrifuge at 300 g for 5 min→discard the supernatant and add 500 ul Trizol to the precipitate. mRNA was extracted from leukocytes using Trizol extraction. mRNA was reverse transcribed into cDNA using the PrimeScript™ RT Master Mix kit from Baoji-Medical Biotechnology (Beijing) Co. The mRNA was diluted 5-fold with enzyme-free water. The primer sequences were designed as follows: LTP1 upstream sequence: 5′-CTGGCGAACAAACACACTGG-3′, downstream: 5′-CACGGTCCGGTTGTAGTTGA-3′; Gapdh upstream sequence: 5′-GACGTCCGGTTGTAGTTGA-3′; Gapdh upstream sequence: 5′-GACGTCCGGTTGTAGTTGA-3′.: 5′-GACAGTCAGCCGCATCTTCT-3′, downstream: 5′-GCGCCCAATACGACCAAATC-3′. The qPCR reaction program was set as follows: 95℃/30 s- 95℃/5 s- 60°C/30 s-Dissosiation stage. To validate the stability of GAPDH expression under hypoxic conditions characteristic of STEMI, we also measured β-actin expression as an alternative reference gene unaffected by hypoxia. The β-actin primer sequences were: forward 5′-CATGTACGTTGCTATCCAGGC-3′, reverse 5′-CTCCTTAATGTCACGCACGAT-3′. Comparative analysis showed consistent results between GAPDH and β-actin normalization (correlation coefficient *r* = 0.92, *P* < 0.001), confirming GAPDH as a reliable reference gene in our experimental context.R esults were analyzed using the 2^−ΔΔ^ CT method.

### Grouping

During the six-month follow-up period, three patients were lost to follow-up, one died of non-cardiac causes, and 92 patients were effectively enrolled. The arterial LRP1 expression values of these 92 STEMI patients were arranged in ascending order and divided into tertiles, resulting in three groups: low LRP1 group (<0.31, *n* = 30), middle LRP1 group (0.31–0.94, *n* = 31) and high LRP1 group (>0.94, *n* = 31).

The tertile-based classification was chosen to ensure balanced group sizes for statistical power while exploring potential non-linear relationships between LRP1 expression and clinical outcomes. Although Youden's index identified an optimal cutoff (0.79) for MACE prediction (see Results), tertiles provide a distribution-based categorization that is less sensitive to cohort-specific thresholds and may better reflect biological variability in LRP1 expression.

### Follow-up

The follow-up time was six months, and the selected patients were followed up regularly at one month, three months, and six months after discharge, including telephone contact, outpatient follow-up, electronic medical record system review, etc. The endpoints of the present study were MACE events during the follow-up period.MACE events include cardiac death, unplanned unstable angina pectoris, nonfatal acute myocardial infarction and acute heart failure, high atrioventricular block, ventricular tachycardia, ventricular fibrillation, and other malignant arrhythmia.

### Statistical analysis

Measurement data were tested for normality and expressed as mean ± standard deviation (x ± s) when normal distribution was met, and a two-sample *t*-test was used for comparison between two independent samples, and analysis of variance was used for comparison between multiple groups; if the normal distribution was not met, it was expressed as median (interquartile spacing) or M (P25, P75), and the comparison between two independent samples was performed using the Mann–Whitney *U*-test, and the multi-sample rank-sum H test was used for comparison between groups; count data were expressed as percentage, and *χ*^2^ test or Fisher's exact test was used for comparison between groups. Comparisons between groups were made using the multi-sample rank and H test; count data were expressed as percentages, and comparisons between groups were made using the *χ*^2^ test or Fisher's exact test. Pearson's correlation analysis or Spearman's rank correlation was used for correlation analysis. The predictive value of LRP1 for MACE events within six months after PCI in STEMI patients was analyzed using the Receiver operating characteristic (ROC) curve. A value of *P* < 0.05 was supposed to be statistically significant. All statistical analyses were performed using SPSS Version 29.0.

## Results

### Comparison of LRP1 expression between control and experimental groups

**Figure 2:**
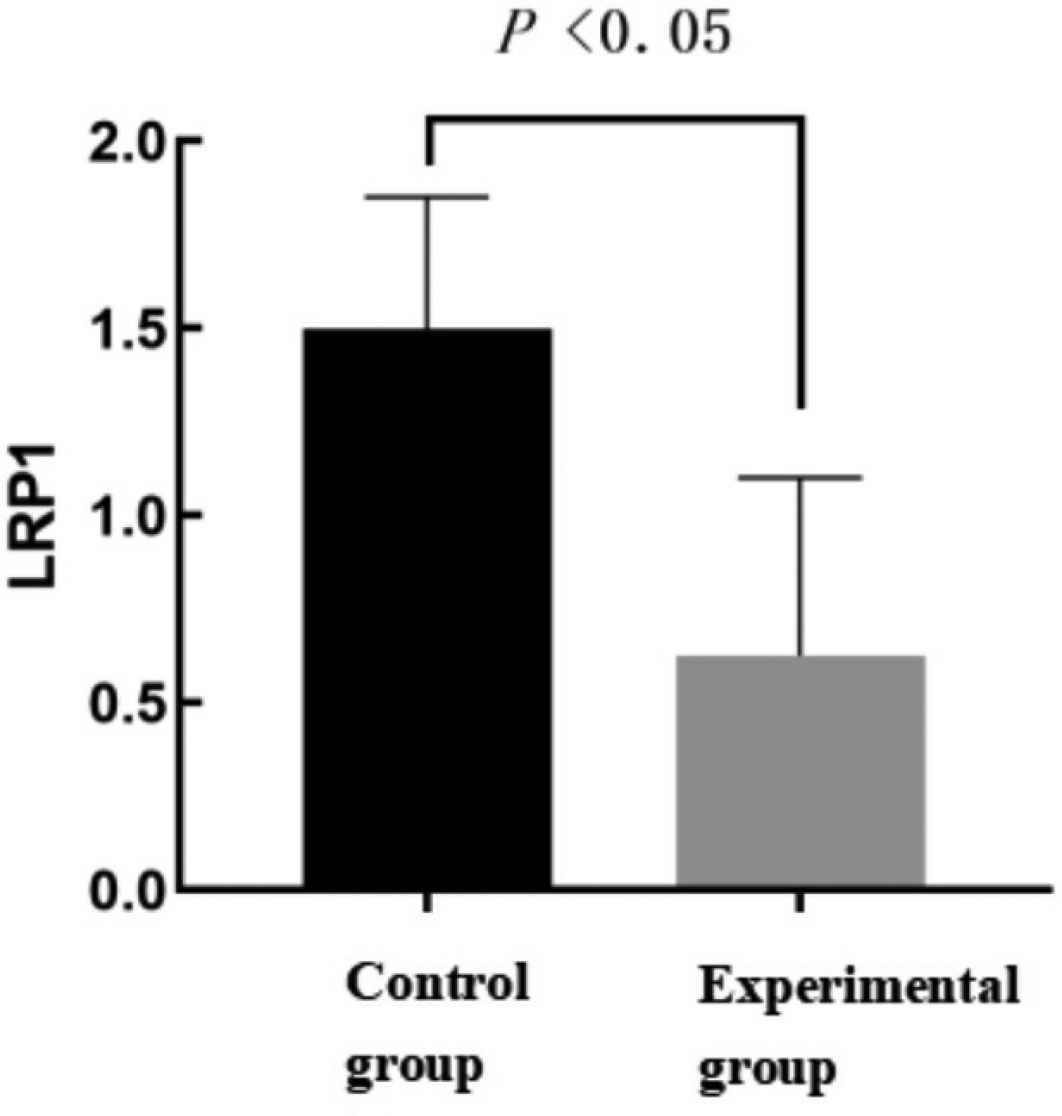
Comparison of LRP1 expression between control and experimental groups.

As shown in Figure 2 and Table 1, the arterial blood LRP1 expression in the control group was 1.5 (0.84,1.85), and that in the experimental group was 0.63 (0.23,1.1), and there was a statistically significant difference in the distribution of overall LRP1 expression between the two groups (*Z* = −3.022, *P* = 0.003).

**Table 1:** Comparison of LRP1 expression between control and experimental groups.

Variable	Control subjects (*n* = 19)	Experimental group (*n* = 92)	Z-value	*P*
LRP1 (arterial)	1.5 (0.84,1.85)	0.63 (0.23,1.1)	−3.022	0.003

### Characteristics of patients in the experimental group

The differences in systolic blood pressure, diastolic blood pressure, mean arterial pressure, cTnT, NT-proBNP, and LVEF values among the three groups were statistically significant (*P* < 0.05). As shown in Table 2.

**Table 2:** Comparison of the results of the general information.

Characteristics	Low LRP1	Middle LRP1	High LRP1	t/*χ*^2^/F value	*P* value
	(*n* = 30)	(*n* = 31)	(*n* = 31)		
Age, years	56.9 ± 14.95	58.48 ± 13.27	61.58 ± 10.39	1.029	0.362
BMI, kg-m^−2^	25.64 ± 3.43	24.78 ± 3.54	25.18 ± 4.19	0.406	0.667
Smoking, *n* (%)	7 (23.3)	10 (32.3)	12 (38.7)	1.682	0.431
Hypertension, *n* (%)	16 (53.3)	16 (51.6)	17 (54.8)	0.065	0.968
Diabetes mellitus, *n* (%)	9 (30)	12 (38.7)	13 (41.9)	0.994	0.608
Systolic pressure, mmHg	120.33 ± 20.26	139.68 ± 22.78^a^	132.32 ± 29.63	4.797	0.011*
Diastolic pressure, mmHg	77.23 ± 10.34	86.94 ± 16.46^a^	81.84 ± 20.69	3.878	0.027*
Mean arterial pressure, mmHg	91.6 ± 13.15	104.52 ± 17.65^a^	98.67 ± 23.09	5.344	0.007*
Coronary multivessel disease, *n* (%)	23 (76.7)	24 (77.4)	25 (80.6)	0.161	0.923
Gensini score	64.5 (46.8, 87.8)	62.5 (42, 98)	62 (44, 104)	0.3	0.985
Beta-blockers, *n* (%)	21 (70)	19 (61.3)	23 (74.2)	1.243	0.537
ACEI/ARB/ARNI, *n* (%)	21 (70)	20 (64.5)	19 (61.3)	0.52	0.771
LDL-C, mmol/L	2.73 ± 0.7	2.67 ± 1.06	2.59 ± 0.87	0.199	0.82
SCr, mmmol/L	70 (64.26, 89.3)	81 (68, 97)	78 (62.4, 93.9)	0.731	0.694
cTnT, ng/ml	2.48 (1.47, 5.42)	3.31 (1.32, 6.2)	5.5 (2.53, 9)^a^	6.48	0.039*
NT-proBNP, pg/ml	708 (246.3, 1362)	1,280 (488, 1897)	1,970 (1000, 2500)^a^	10.49	0.005*
hs-CRP, mg/L	10 (3.95, 22.75)	10 (3.5, 14.5)	13 (6, 25)	4.07	0.131
LV ejection fraction, (%)	58 (55, 59.25)	56 (54, 58)	55 (50, 58)^a^	7.173	0.028*

* Indicates statistically significant differences among the three groups.

^a^ Indicates *P* < 0.05 compared with the low LRP1 group; LRP1 expression levels were categorized using tertile cutoff points: Low (<0.31, 33rd percentile), Middle (0.31–0.94, 33rd to 67th percentile), and High (>0.94, >67th percentile). These thresholds were determined based on the distribution of LRP1 expression values in the study population (*n* = 92).

### Follow-up outcomes

Among the 92 effectively enrolled patients, 22 patients with MACE occurred, accounting for 23.9% of the total number of cases, of which eight were admitted with recurrent angina pectoris, two were admitted with recurrent myocardial infarction, 6 were patients with acute heart failure, 2 with malignant arrhythmia, and 4 with cardiac death. The incidence of MACE was significantly higher in the high LRP1 group than in the low LRP1 group (*P* < 0.05). There was no statistical difference among the three groups in the composition ratio of new-onset angina, myocardial infarction, acute left heart failure, malignant arrhythmia, and cardiac death (*P* < 0.05). Detailed results of the analysis are shown in Table 3.

**Table 3:** MACEs in the three groups (cases, %).

MACE events	Low LRP1 group	Middle LRP1 group	High LRP1 group	*χ*^2^ value	*P* value
	(*n* = 30)	(*n* = 31)	(*n* = 31)		
Angina pectoris, myocardial infarction	1 (3.3)	4 (12.9)	5 (16.1)	4.97	0.083
Acute left heart failure	1 (3.3)	1 (3.2)	4 (12.9)	2.915	0.233
arrhythmia	0 (zero)	1 (3.2)	1 (3.2)	1.797	0.518
cardiac death	0 (zero)	1 (3.2)	3 (9.7)	2.933	0.319
add up the total	2 (6.7)	7 (22.6)	13 (41.9)*	10.530	0.005

* *P* < 0.05 compared to low LRP1 group.

### Correlation analysis of LRP1 with Nt-proBNP, cTnT, LVEF values, and other indicators

Spearman rank correlation analysis showed that LRP1 was positively correlated with NT-proBNP, cTnT, systolic blood pressure, and mean arterial pressure and negatively correlated with LVEF values. As shown in Table 4.

**Table 4:** Correlation analysis of LRP1 with other indicators.

Variables	LRP1	
	r	*P*-value
NT-proBNP	0.349	<0.001
LVEF (%)	−0.285	0.006
cTnT	0.328	0.001
Systolic pressure	0.222	0.034
mean arterial pressure	0.230	0.027

### Analysis of the predictive value of LRP1 for MACE events within six months after PCI in STEMI patients

**Figure 3:**
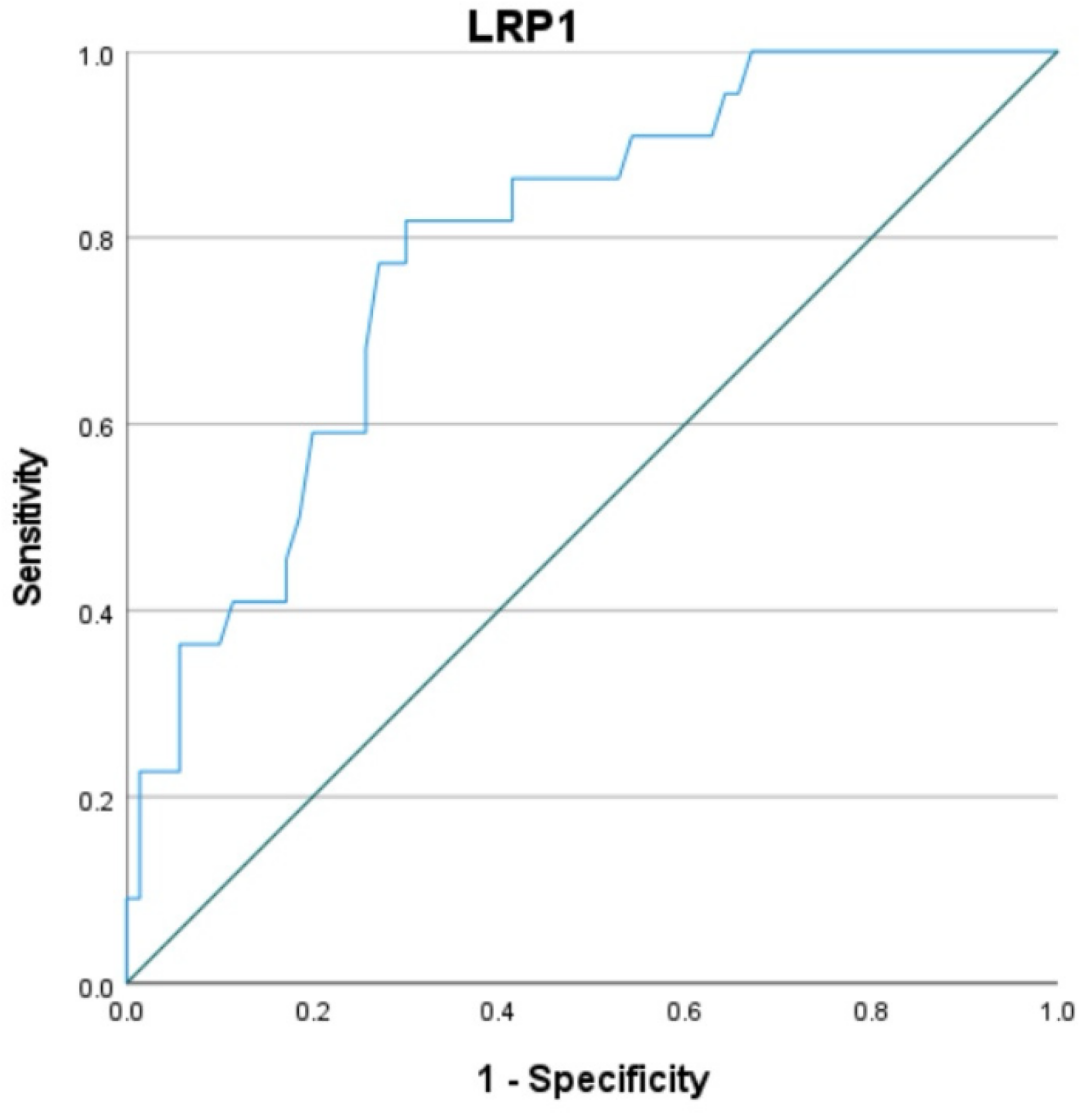
ROC curve of LRP1 predicting prognosis of AMI patients after PCI.

The predictive value of LRP1 expression level on the occurrence of MACE events in STEMI patients after emergency PCI was explored using the ROC curve, with MACE events occurring six months after PCI as the observation endpoint. As determined by the Youden index, LRP1, with a cutoff value of 0.79, had a sensitivity of 81.8% and a specificity of 70% for predicting MACE. The area under the ROC curve (AUC) was 0.789 (95% CI: 0.688–0.890, *P* = 0.000), as shown in Figure 3.

Multivariate analysis for MACE prediction To evaluate whether LRP1 is an independent predictor of MACE, we performed multivariate logistic regression analysis. Variables with *P* < 0.05 in univariate analysis and clinically relevant factors were included in the multivariate model. The results showed that high LRP1 expression (>0.79), elevated NT-proBNP, reduced LVEF, and elevated cTnT were independent predictors of 6-month MACE in STEMI patients after PCI. The detailed results are presented in Table 5.

**Table 5:** Univariate and multivariate logistic regression analysis for 6-month MACE in STEMI patients after PCI.

Variables	Univariate analysis			Multivariate analysis		
	95% CI	*P* value	OR	95% CI	*P* value	OR
LRP1 > 0.79	3.86	1.74–8.52	0.001	2.93	1.28–6.71	0.011
NT-proBNP > 1,500 pg/ml	2.95	1.38–6.29	0.005	2.41	1.09–5.33	0.030
LVEF < 55%	2.73	1.25–5.94	0.012	2.15	1.03–4.48	0.041
cTnT > 5.0 ng/ml	2.58	1.19–5.61	0.017	1.98	0.94–4.17	0.072
Systolic pressure > 140 mmHg	1.85	0.86–3.97	0.115			
Mean arterial pressure > 100 mmHg	1.67	0.77–3.62	0.193			
Age > 65 years	1.54	0.71–3.35	0.276			
Multivessel disease	1.48	0.68–3.21	0.323			

### Subgroup analyses

To further evaluate the impact of different clinical factors on outcomes, we conducted subgroup analyses based on DAPT regimens, statin types, and gender.

### DAPT subgroup analysis

Patients were divided based on their DAPT regimens (ticagrelor + aspirin vs. clopidogrel + aspirin). The incidence of MACE was compared across LRP1 tertiles within each DAPT subgroup. As shown in Table 6, the association between higher LRP1 levels and increased MACE risk remained consistent regardless of DAPT type, though the ticagrelor group showed a numerically lower MACE rate.

**Table 6:** Incidence of MACE across LRP1 tertiles stratified by DAPT regimens.

DAPT type	Group	Total cases	MACE events	MACE rate (%)	*P* value
Ticagrelor + Aspirin	Low LRP1	16	1	6.3	0.008
	Middle LRP1	17	3	17.6	
	High LRP1	16	6	37.5	
Clopidogrel + Aspirin	Low LRP1	14	1	7.1	0.012
	Middle LRP1	14	4	28.6	
	High LRP1	15	7	46.7	

### Statin subgroup analysis

We analyzed MACE rates across LRP1 tertiles stratified by statin intensity (high-intensity vs. moderate-intensity). As presented in Table 7, the prognostic value of LRP1 was maintained in both statin subgroups, with high-intensity statin users showing relatively lower MACE rates.

**Table 7:** Comparison of MACE rates among LRP1 tertiles according to statin therapy intensity.

Statin intensity	Group	Total cases	MACE events	MACE rate (%)	*P* value
High-intensity	Low LRP1	18	1	5.6	0.015
	Middle LRP1	19	4	21.1	
	High LRP1	18	7	38.9	
Moderate-intensity	Low LRP1	12	1	8.3	0.009
	Middle LRP1	12	3	25.0	
	High LRP1	13	6	46.2	

### Gender-based subgroup analysis

Despite the limited number of female patients in our cohort, we performed a gender-based subgroup analysis to explore potential sex-specific differences in the prognostic value of LRP1. Table 8 shows the results stratified by gender.

**Table 8:** Gender-specific analysis of MACE occurrence across LRP1 tertiles in STEMI patients.

Gender	Group	Total cases	MACE events	MACE rate (%)	*P* value
Male	Low LRP1	26	2	7.7	0.007
	Middle LRP1	27	6	22.2	
	High LRP1	27	11	40.7	
Female	Low LRP1	4	0	0.0	0.038*
	Middle LRP1	4	1	25.0	
	High LRP1	4	2	50.0	

* *P* < 0.05 compared with low LRP1 group.

## Discussion

LRP1 is a multifunctional transmembrane receptor that plays a role not only in lipoprotein metabolism but also in the progression of atherosclerosis, myocardial ischemia-reperfusion, and ventricular remodeling. Drevinge C et al. found that LRP1 expression was significantly up-regulated in infarct areas with reversible and irreversible heart damage in a porcine model of ischemia-reperfusion (8). In a STEMI mouse model, researchers observed that LRP1 gene expression and LRP1 protein levels were low in the heart infarction area during the inflammatory reaction stage (day one after STEMI), whereas in the fibrotic stage (days 10 and 21 after STEMI), the expression of LRP1 gene and protein levels in myocardial tissue and fibroblasts and the infarction area were significantly increased. Additionally, the expression of proline-rich tyrosine kinase two phosphorylation (pPyk2) and matrix metalloproteinases 9 (MMP-9) was strongly up-regulated, with myocardial LRP1 strongly coexisting with pPyk2 and MMP-9 to co-regulate cardiac remodeling after STEMI (9). Furthermore, our study measured LRP1 expression in leukocytes from arterial blood samples, with monocytes likely being the primary cellular source. This is supported by previous research showing that LRP1 is predominantly expressed in monocytes and plays a crucial role in atherosclerosis progression. A recent study demonstrated that decreased LRP1 expression in pro-inflammatory monocytes is associated with subclinical atherosclerosis (10), which may explain the reduced LRP1 levels we observed in early STEMI patients. The downregulation of monocyte LRP1 expression could potentially serve as an early marker of acute coronary events.

In the present study, comparison of LRP1 expression levels in arterial blood between the experimental and control groups revealed that the expression level of LRP1 in early STEMI patients was lower than that in the control group overall, which is largely consistent with the above research results. The 6-month follow-up after PCI showed significant differences in the incidence of MACE among the three groups of low, middle, and high LRP1. As LRP1 expression levels increased, the incidence of MACE was higher. According to ROC curve analysis, when the expression level of LRP1 was greater than 0.79, the probability of MACE occurrence was higher within six months after PCI, with a sensitivity of 81.8% and a specificity of 70%. These results suggest that arterial LRP1 levels at the initial stage of STEMI may have clinical value in predicting the occurrence of MACE in STEMI patients. While our study demonstrates an association between LRP1 levels and MACE, the precise biological mechanisms underlying this relationship warrant further discussion. LRP1 is known to play a complex role in various cellular processes, including inflammation, cell survival, and extracellular matrix remodeling. In the context of STEMI, it is plausible that altered LRP1 expression may reflect the extent of myocardial injury, inflammatory response, and the initiation of adverse remodeling processes. Specifically, the observed upregulation of LRP1 in patients experiencing higher MACE rates could indicate a compensatory response or an attempt to initiate tissue repair. However, in the setting of severe myocardial damage and ongoing inflammation, this response might be insufficient to prevent adverse events, or even potentially contribute to detrimental remodeling. Further studies, perhaps involving *in vitro* experiments or the analysis of specific signaling pathways, are necessary to elucidate the precise mechanisms by which LRP1 influences the development of MACE after STEMI.

In this study, Spearman rank correlation analysis showed that LRP1 was positively correlated with NT-proBNP and cTnT and negatively correlated with LVEF value, indicating that arterial LRP1 could reflect myocardial ischemia degree, ventricular wall tension, and cardiac systolic function in the early STEMI period to a certain extent. In addition, LRP1 was positively correlated with systolic pressure and mean arterial pressure. Studies have shown that cardiovascular risk factors such as hypertension and hyperlipidemia can also induce upregulation of LRP1 to varying degrees, jointly leading to overexpression of LRP1 in advanced atherosclerotic plaques (11, 12). GamboaR et al. also found that the expression levels of LRP1mRNA and LRP1 protein in monocytes of hypertensive patients were significantly up-regulated (13), which was basically consistent with the results of this study. However, no correlation between LRP1 and blood lipid and other indicators was found in this study, which may be due to the dual reasons of the past use of statins in some patients and the low level of LRP1 expression in early STEMI. Several studies have confirmed that upregulation of LRP1 expression can protect cardiomyocytes by promoting Akt and ERK1/2-dependent survival pathways through binding with the serine protease inhibitor complex (14, 15). However, due to the limited effects of enzyme inhibitor complex and LRP1 pro-survival signal triggering in the early stage of STEMI, it is not sufficient to combat the excessive inflammatory response in the early infarction area, so serine protease inhibitors (plasma-derived AAT, SP16, etc.), which act as LRP1 agonists, are more commonly developed and used in STEMI ischemia-reperfusion. In animal experiments, SP16 applied within 30 min of reperfusion and binding earlier with LRP1 can provide rapid phosphorylation of Akt and down-regulation of NF-kB inflammatory signals, inhibit programmed death of cardiomyocytes, and thus exert anti-inflammatory and more powerful cardiomyocyte protection (5, 16). In a small sample clinical trial, STEMI patients treated with SP16 showed no adverse reactions after one year of follow-up, with CRP, CK-MB and other indicators significantly decreased, and LVEF values significantly improved from baseline levels (3, 15–17). However, large-scale clinical trials are still necessary to confirm the safety and effectiveness of exogenous serine protease inhibitors.

Several limitations of this study should be acknowledged. Firstly, the relatively small sample size of 96 STEMI patients may limit the generalizability of our findings. This is especially true considering the heterogeneous nature of STEMI, encompassing varying degrees of myocardial damage, patient comorbidities, and treatment approaches. Therefore, our conclusions should be interpreted with caution, and further validation in larger, multi-center cohorts is crucial to confirm our results and establish the robustness of LRP1 as a prognostic marker. Secondly, the 6-month follow-up period is relatively short for assessing long-term cardiovascular outcomes. While it allowed us to evaluate the short-term predictive value of LRP1, it may not fully capture the complexities of cardiac remodeling and its impact on longer-term prognosis. Future research should aim to extend the follow-up period to several years to gain a more complete understanding of the prognostic implications of LRP1 in STEMI patients. Third, while our study evaluated the occurrence of MACE within six months, the exact timing of events was not systematically recorded. This precluded time-to-event analyses such as Kaplan–Meier survival curves, which could provide additional insights into the temporal relationship between LRP1 levels and adverse outcomes. Future studies should incorporate detailed event timing to enable survival analysis.

Furthermore, the integration of LRP1 measurement into current clinical practice is a critical consideration. While our results suggest a potential clinical utility for LRP1 assessment, several practical hurdles need to be addressed. Issues related to cost, the availability of reliable assays, and the requirement for specialized equipment are all important factors that need to be addressed. Moreover, the logistical challenges of obtaining and processing blood samples in an emergency setting may also present practical obstacles to the widespread adoption of LRP1 measurement. Additional investigations, including cost-effectiveness analyses and the development of point-of-care LRP1 assays, are needed to facilitate the smooth and effective translation of our findings into clinical practice. This includes exploring the potential for using existing laboratory infrastructure to perform LRP1 assessments and identifying strategies to minimize the cost and time requirements associated with these measurements.

In this study, only blood samples before vascular opening were collected, and the changes in LRP1 expression at different stages were not dynamically monitored, only LRP1 levels on white blood cells were measured, and specific cells and their subtypes were not accurately identified, and in the future, dynamic monitoring of LRP1 changes is needed to explore the functions and effects of LRP1 on different cell subtypes in different stages of STEMI.In addition, PCSK9 can reduce the LRP1 receptor, and whether PCSK9 inhibitors can play the role of LRP1 agonists needs further large-scale studies (18).

In conclusion, our study suggests that LRP1 expression appears to be an independent predictor of MACE in STEMI patients and may have prognostic value for short-term outcomes following PCI. However, further studies are essential to elucidate the precise biological mechanisms underlying the observed association, validate our findings in larger, more diverse cohorts, extend the follow-up duration, and establish the clinical feasibility of LRP1 measurement in routine practice.

## Data Availability

The raw data supporting the conclusions of this article will be made available by the authors, without undue reservation.
